# Cucurbitacin I blocks cerebrospinal fluid and platelet derived growth factor-BB stimulation of leptomeningeal and meningioma DNA synthesis

**DOI:** 10.1186/1472-6882-13-303

**Published:** 2013-11-04

**Authors:** Mahlon D Johnson, Mary J O’Connell, Kevin Walter

**Affiliations:** 1 Division of Neuropathology, Department of Pathology and Laboratory Medicine, University of Rochester School of Medicine and Dentistry, Box 626, 601 Elmwood Ave., Rochester, NY 14642, USA; 2 Division of Neurosurgery, University of Rochester School of Medicine and Dentistry, Rochester, New York, USA

## Abstract

**Background:**

Currently, there are no consistently effective chemotherapies for recurrent and inoperable meningiomas. Recently, cucurbitacin I (JSI-124), a naturally occurring tetracyclic triterpenoid compound used as folk medicines has been found to have cytoxic and anti-proliferative properties in several malignancies thru inhibition of activator of transcription (STAT3) activation. Previously, we have found STAT3 to be activated in meningiomas, particularly higher grade tumors.

**Methods:**

Primary leptomeningeal cultures were established from 17, 20 and 22 week human fetuses and meningioma cell cultures were established from 6 World Health Organization (WHO) grade I or II meningiomas. Cells were treated with cerebrospinal fluid from patients without neurologic disease. The effects of cucurbitacin I on cerebrospinal fluid stimulation of meningioma cell DNA synthesis phosphorylation/activation of JAK1, STAT3, pMEK1/2, p44/42MAPK, Akt, mTOR, Rb and caspase 3 activation were analyzed in human leptomeningeal and meningioma cells.

**Results:**

Cerebrospinal fluid significantly stimulated DNA synthesis in leptomeningeal cells. Co-administration of cucurbitacin I (250 nM) produces a significant blockade of this effect. Cucurbitacin I alone also produced a significant reduction in basal DNA synthesis. In grade I and II meningiomas, cerebrospinal fluid also significantly stimulated DNA synthesis. Co-administration of cucurbitacin I (250 nM) blocked this effect.

In the leptomeningeal cultures, cerebrospinal fluid stimulated STAT3 phosphorylation but not p44/42MAPK, Akt or mTOR. Cucurbitacin I had no effect on basal STAT3 phosphorylation but co-administration with cerebrospinal fluid blocked cerebrospinal fluid stimulation of STAT3 phosphorylation in each. In the grade I meningiomas, cerebrospinal fluid stimulated phosphorylation of STAT3 and decreased MEK1/2 and cucurbitacin I had no effect on basal STAT3, p44/42MAPK, Akt, JAK1, mTOR, or Rb phosphorylation. In the grade II meningiomas, cerebrospinal fluid stimulated STAT3 phosphorylation in all and reduced phosphorylation of MEK1/2 in all and p44/42MAPK in one. Cucurbitacin I had no effect on basal phosphorylation of STAT3 but reduced phorphorylated p44/42 MAPK in 2 grade II meningioma cells lines.

**Conclusions:**

These studies raise the possibility that cucurbitacin I might have value as an adjunct chemotherapy. Additional studies are warranted to evaluate the effects of cucurbitacin I on meningiomas in vivo.

## Background

The lack of effective chemotherapies compromises management of recurrent or inoperable meningiomas [1,2]. Approximately 12% of intracranial world health organization (WHO) grade I meningiomas recur within 5 years of gross total resection [3,4]. Intracranial, WHO grade II, tumors may recur with a frequency as high as 29-41% within 5 years after gross total resection [5,6]. Recurrence rates of skull base meningiomas are even higher [7,8]. In these cases effective chemotherapies have not been established and recent promising treatments have proven suboptimal [9-12]. In most recurrences, management includes radiotherapy but this may be less effective [13] than in cases of de novo meningiomas where radiation can produce a 93% control at 5 years and 87% at 15 years [14]. In a recent study of Gamma Knife-treated skull base meningiomas, followed for up to 103 months, 33% exhibited variable regression, 64% were stabilized and 6% showed progression [15]. However, stereotactic radiosurgery produced only a 49% progression free survival in atypical, WHO grade II meningiomas and is ineffective in treatment of anaplastic meningiomas [15]. Moreover in some studies, radiotherapy as an initial treatment was less effective in large meningiomas greater than 5 cm^3^[16].

Meningioma growth is regulated by complex interactions between multiple mitogenic/anti-apoptotic signaling pathways [17]. Previously, we have found that several of these pathways such as the MAPK kinase kinase-MAPK kinase-mitogen activated protein kinase (Raf-1-MEK-1-MAPK) pathway, phosphoinositide 3 kinase-protein kinase B/Akt-proline rich-Akt substrate 40-mTOR (PI3K-PKB/Akt-PRAS40-mTOR) pathway and phospholipase C-gamma pathways are activated in WHO grade I meningiomas [18-20]. The janus tyrosine kinase (JAK)-signal transducer and activator of transcription (STAT3) pathway, and/or STAT3 might also be involved [21-24]. These pathways may be activated by autocrine or paracrine growth factor/cytokine stimulation of meningioma cells rather than constitutive activation of growth factor receptors or signal transduction pathways [16,23]. Autocrine production of many mitogenic cytokines and their presence in the cerebrospinal fluid have been established [16,17,23,25].

**Figure 1:**
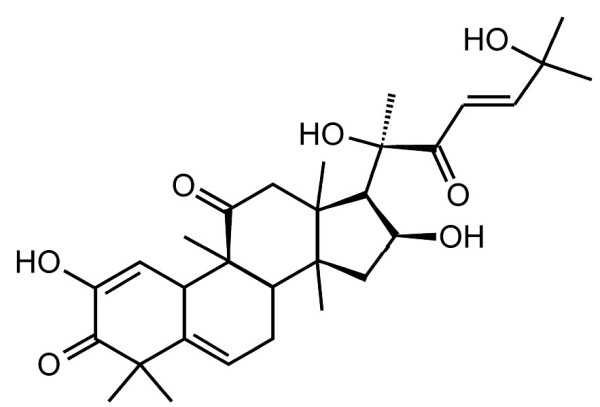
**Structure of cucurbitacin I.** Cucurbitacin I is a tetracyclic triterpene compound that has been used in Chinese, Indian and South American folk medicine.

Cucurbitacins are a family of naturally occurring tetracyclic triterpenoid compounds that have been used as folk medicines in China, India, Peru and Brazil for centuries [26-29] (Figure 1). Six forms have been recognized. Cucurbitacin I, (JSI-124) [26] has recently been found to have cytoxic and anti-proliferative properties in adenocarcinoma cells [29] nasopharyngeal carcinoma cells and xenographs [30], glioblastomas [31] anaplastic large cell lymphoma [32] and non small cell carcinoma [33]. Initial studies suggested that this was largely thru inhibition of the janus kinase-3/signal trasnducer and activator of transcription-3 (JAK/STAT3) activation [26]. Cucurbitacin may also inhibit tumor cell invasion [30] and has antiinflammatory properties [34,35].

Previously we have found that cerebrospinal fluid stimulation of leptomeningeal and meningioma cell proliferation is associated, in part, with activation of STAT3. In search of a STAT3 inhibitor, we tested cucrbitacin I on STAT3 activation in one leptomeningeal and meningioma culture [23]. In the present study, to characterize cucurbitacin I, we evaluated its effects on proliferation and growth regulatory pathways in numerous human leptomeningeal, grade I and II meningioma cell cultures. To mimic the in vivo milieu, we stimulated cells with cerebrospinal fluid from patients found to have no neurological disease and platelet derived growth factor BB (PDGF-BB), a known mitogen for meningioma cells [19,20,23].

## Methods

### Human leptomeningeal and meningioma cell cultures

Primary leptomeningeal cultures were established from de-identified postmortem leptomeninges from 17, 20 and 22 week human fetuses with University of Rochester Institutional Review Board approval and postmotrm exception but prior autopsy/surgical maternal consent for use of the tissue. Remnant, excess, discarded tissue/meningioma cells were also collected (mean age 61 years; 4 females) with University of Rochester Institutional Review Board approval and surgical consent for use of tissue. M1-M3 were WHO grade I meningothelial meningiomas from the clivus, sphenoid wing or frontal lobe and all had detectable Merlin protein. M4-M6 included a WHO grade II meningothelial, transitional and microcystic meningioma from the frontal lobe and the 2 analyzed also had detectable Merlin protein. These cells were maintained in DMEM with 10% fetal bovine serum (FBS) which is insufficient for normal glia and endothelial cell survival and proliferation. The cells used were screened for features of leptomeningeal and meningioma cells following procedures described previously including detection of a leptomeningeal marker, epithelial membrane antigen (EMA) [36]. For experiments, only early passage i.e. passage 2 to 5 were used on meningioma cell cultures that showed EMA and variable desmoplakin immunoreactivity by immunocytochemistry and Merlin protein [37,38]. For immunocytochemical characterization, meningioma cell cultures were plated onto 2 well microscope slides (Nalgene NUNC Int., Rochester, NY) for 1 day in DMEM with 10% FBS then fixed in formalin. Immunocytochemistry was performed using a mouse monoclonal antibody against epithelial membrane antigen (EMA, prediluted) (DAKO, Carpenteria, CA) and anti-desmoplakin (1:200; Abcam Inc., Cambridge MA) using the streptavidin-biotin-horseradish peroxidase method. The presence or loss of Neurofibromatosis-2 gene product Merlin was assessed by western blot (data not shown, 37, 38).

### Human cerebrospinal fluid from patients without neurologic disease

Remnant, discarded lumbar cerebrospinal fluid was retrieved from samples collected at the University of Rochester Medical Center (Rochester, NY) and sent to the Dept. of Pathology for analysis between March, 2009 and September, 2012. As discarded remnant tissue, it was exempted by the University of Rochester Institutional review board from patient consent forms beyond standard patient consent forms for tissue use. Samples from patients found to be free of neurological disease that had cytopathology analysis showing no erythrocytes or increased lymphocytes and normal indices were classified as “normal”. In patients who had cerebrospinal fluid collected as part of staging protocol for a peripheral lymphoma, only cerebrospinal fluid from those with no cells by cytology and no neurologic disease were used. Due to limited volume, in some cases multiple samples were combined as pools to achieve some uniformity and enough volume to treat large numbers of cells in T75 flasks. Samples were initially frozen at -17°C then stored at -80°C.

### Effects of cucurbitacin I on cerebrospinal fluid stimulation of meningioma cell DNA synthesis

Confluent cells from human 17, 20 and 22 week fetal leptomeninges (L1-L3), 3 WHO grade I (M1 -M3) and 3 WHO grade II (M4- M6) were plated in 96 well plates, serum deprived overnight then treated with serum free DMEM, undiluted cerebrospinal fluid, DMEM and cucurbitacin I (250 nM) or cerebrospinal fluid with cucurbitacin (250 nM, Calbiochem, CA) for 72 hrs. This dose was chosen based on the dose response curves and previous literature using doses as high as 10 uM [26] or lower [30,31]. Cells were subsequently analyzed in 6 replicates by CyQUANT Cell Proliferation assay (Invitrogen, Carlsbad, CA). Samples were analyzed with a fluorescence microplate reader with filters appropriate for ~480 nm excitation and ~520 nm emission maxima. Differences between treatment groups were analyzed by unpaired, two-tailed T-tests (In Stat, Sigma, St. Louis).

### Dose response effects of cucurbitacin I on cerebrospinal fluid and PDGF-BB stimulation of human leptomeningeal and meningioma cell DNA synthesis

For analysis of effects of various doses of cucurbitacin on cerebrospinal fluid stimulation of a grade I (M1) meningioma were also plated in 96 well plates, serum deprived overnight then treated with serum free DMEM, DMEM with cerebrospinal fluid with or without cucurbitacin I 25, 75 or 250 nM for 72 hrs. Cells were subsequently analyzed in 6 replicates by CyQUANT Cell Proliferation assay (Invitrogen, Carlsbad, CA). Differences between treatment groups were analyzed by unpaired, two-tailed T-tests (In Stat, Sigma, St. Louis).

For analysis of effects on PDGF-BB, cells of 20wk fetal leptomeninges, (L2) were also plated in 96 well plates, serum deprived overnight then treated with serum free DMEM, DMEM with recombinant human PDGF-BB (10 ng/ml, R and D, Minneapolis, MN) with or without cucurbitacin I 50, 100 or 200 nM for 72 hrs. Cells were subsequently analyzed as above.

### Effects of cerebrospinal fluid and cucurbitacin I on meningioma cell JAK, STAT3, MEK1/2, p44/42MAPK, Akt, mTOR, and Rb activation

Confluent cells from 17, 20 and 22 week primary fetal leptomeningeal cell cultures (L1-3), 2 WHO grade I (M2 -M 3) and 2 WHO grade II primary meningioma cultures (M 4 and M5) were serum deprived overnight then treated with serum free DMEM, cerebrospinal fluid without or with 250 nM cucurbitacin I. Lysates of the cells were then analyzed by Western blots.

For Western blots, meningioma cells were scrapped in RIPA Lysis Buffer (Upstate Biotechnology) with 1:100 Protease Inhibitor Cocktail (Sigma) then vortexed vigorously and frozen at –85°C. Protein concentrations were quantified using a Bradford assay (BioRad Protein Assay reagent), then 10–35 ug protein from each was loaded on 7.5% acrylamide gel then transferred to 0.45 um nitrocellulose membrane. The membrane was blocked 1 hour in 5% milk in Tris-Cl buffer with Tween 20 then reacted with an affinity purified primary antibody overnight at 4°C. This was followed by horseradish peroxidase conjugated secondary antibody treatment. Detection was achieved with Western Lightening (Perkin Elmer) on Xomat film (Kodak).

Western blots were analyzed with monoclonal antibodies to STAT3, phospho-STAT3 phosphorylated at tyrosine 705, JAK, phospho-JAK1 phosphorylated at tyrosine 1022/1023, phosphso-MEK1/2 phosphorylated at serine 221, MAPK 44/42, phospho-MAPK 44/42 phosphorylated at threonine 180/tyrosine 182, Akt, phospho-Akt phosphorylated at threonine 308, phospho-mTOR phosphorylated at serine 2448, Rb and p-Rb phosphorylated at serine 608 (all from Cell Signaling, Beverly MA). Loading was assessed with an antibody to actin.

### Effects of cucurbitacin I on caspase 3 activation in meningiomas

To screen for Caspase 3 activation by ARP cleavage, near confluent cells from cultures of 2 leptomeninges (L1 and L3) and a WHO grade I (M1) primary meningioma culture were treated with serum free DMEM with and without cucurbitacin I (250 nM) or cerebrospinal fluid with or without cucurbitacin (250 nM). Cucurbitacin I’s effects on apoptosis was measured by Caspase 3 activation. Poly (ADP ribose) polymerase 1 (PARP) fragmentation is an indirect, semiquatitative measurement of caspase 3 activation and apoptosis. Cleavage of the 117-kDa PARP into the 85 kDa product was assessed by Western blot with antibody to PARP -1 (Santa Cruz, Biotechnology, Santa Cruz, CA) and blotting procedures detailed above.

## Results

### Effects of cucurbitacin I on cerebrospinal fluid stimulation of meningioma cell DNA synthesis

**Figure 2:**
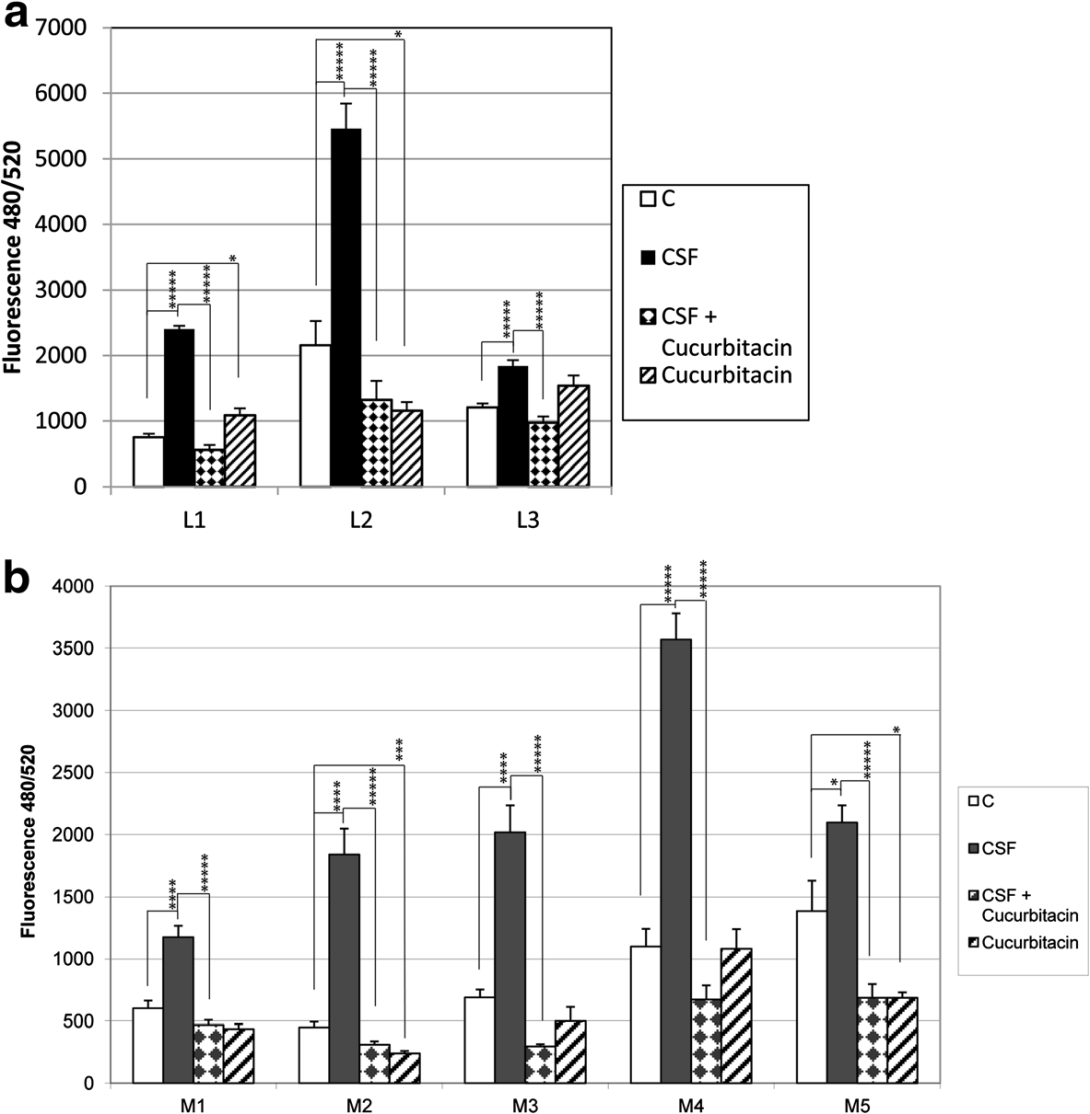
**Effects of cucurbitacin I on cerebrospinal fluid stimulation of leptomeningeal and meningioma cell DNA synthesis. a.** Cells from 17 (L1), 20 (L2) and 22 (L3) wk fetal leptomeninges (L), were treated with CSF without or with cucurbitacin I (250 nM) for 72 hrs. CyQUANT analysis of DNA measured with a fluorescence microplate reader with filters appropriate for ~480 nm excitation and ~520 nm emission maxima. Changes in DNA fluorescence correlate with cell proliferation. C = control, CSF = cerebrospinal fluid. * P < 0.05, *** P < 0.005, **** P < 0.001, ***** P < 0.0005. **b.** Cells from WHO grade I (M1-M3) and WHO grade II (M4-M5) meningiomas were treated as above. * P < 0.05, *** P < 0.005, **** P < 0.001, ***** P < 0.0005.

In the leptomeningeal cells, cerebrospinal fluid significantly stimulates DNA synthesis (p < 0.005). Co-administration of cucurbitacin I (250 nm) produces a significant blockade of this effect (p <0.0005). Cucurbitacin I alone also produced a significant reduction in basal DNA synthesis in L2 (p < 0.05) (Figure 2a). In WHO grade I meningiomas, cerebrospinal fluid significantly stimulated DNA synthesis in M1 – M3 (p < 0.001). Co-administration of cucurbitacin I (250 nm) blocked this effect (p < 0.0005). In M2, cucurbitacin I alone also significantly reduced basal synthesis (p < 0.005). In WHO grade II meningiomas, cerebrospinal fluid significantly stimulated DNA synthesis in M4 and M5 (p < 0.001). Co-administration of cucurbitacin (250 nM) significantly blocked this effect (p < 0.0005). In M5, cucurbitacin reduced basal DNA synthesis (p < 0.05) (Figure 2b).

**Figure 3:**
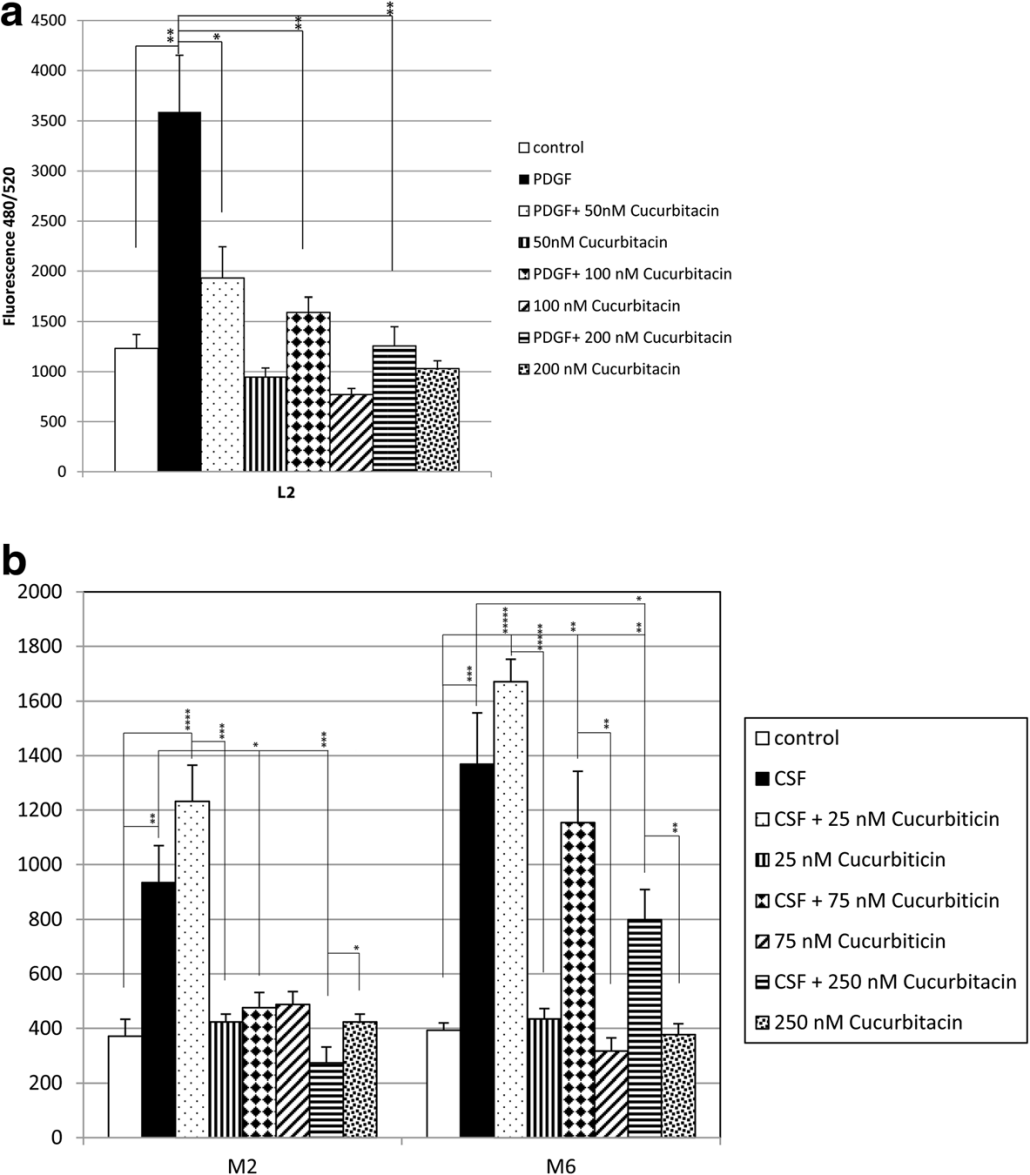
**Cucurbitacin I dose response curves. a.** Effects of cucurbitacin I on PDGF-BB stimulation of human leptomeningeal cell DNA synthesis. Fetal leptomeningeal cells (L2) were treated with PDGF-BB without or with cucurbitacin I (50, 100 or 200 nM) for 72 hrs. CyQUANT analysis of DNA measured by fluorescence at 480/520 nm. Changes in DNA fluorescence correlate with cell proliferation. PDGF = Platelet derived growth factor-BB. * P < 0.05, *** P < 0.005, ***** P < 0.001. **b.** Effects of cucurbitacin on cerebrospinal fluid stimulation of meningioma cell DNA synthesis. WHO grade I (M2) and grade II (M6) meningioma cells were treated with CSF without or with cucurbitacin (25 nM, 75 nM or 250 nM) for 72 hrs. CyQUANT analysis of DNA measured by fluorescence at 480/520 nm. Changes in DNA fluorescence correlate with cell proliferation. CSF = cerebrospinal fluid * P < 0.05, *** P < 0.005, **** P < 0.001, ***** P < 0.0005.

Cucurbitacin I also produced a dose dependent reduction in cerebrospinal fluid stimulation in a grade I meningioma (M2) and l grade 2 (M6). Cucurbitacin I (25 nM) had no effect on cerebrospinal fluid stimulation of DNA synthesis while 75 nM and 250 nM significantly reduced this stimulation (Figure 3b).

### Effects of cucurbitacin on PDGF-BB stimulation of human leptomeningeal and meningioma cell DNA synthesis

In L2, cucurbitacin I produced a dose dependent reduction in PDGF-BB stimulated DNA synthesis at 50 nM (p = 0.005) 100 nM (p = 0.01) and 200 nM (p = 0.01) doses (Figure 3a).

### Effects of cerebrospinal fluid and cucurbitacin I on meningioma cell JAK, STAT3, MEK1/2, p44/42MAPK, Akt, mTOR, and Rb activation

**Figure 4:**
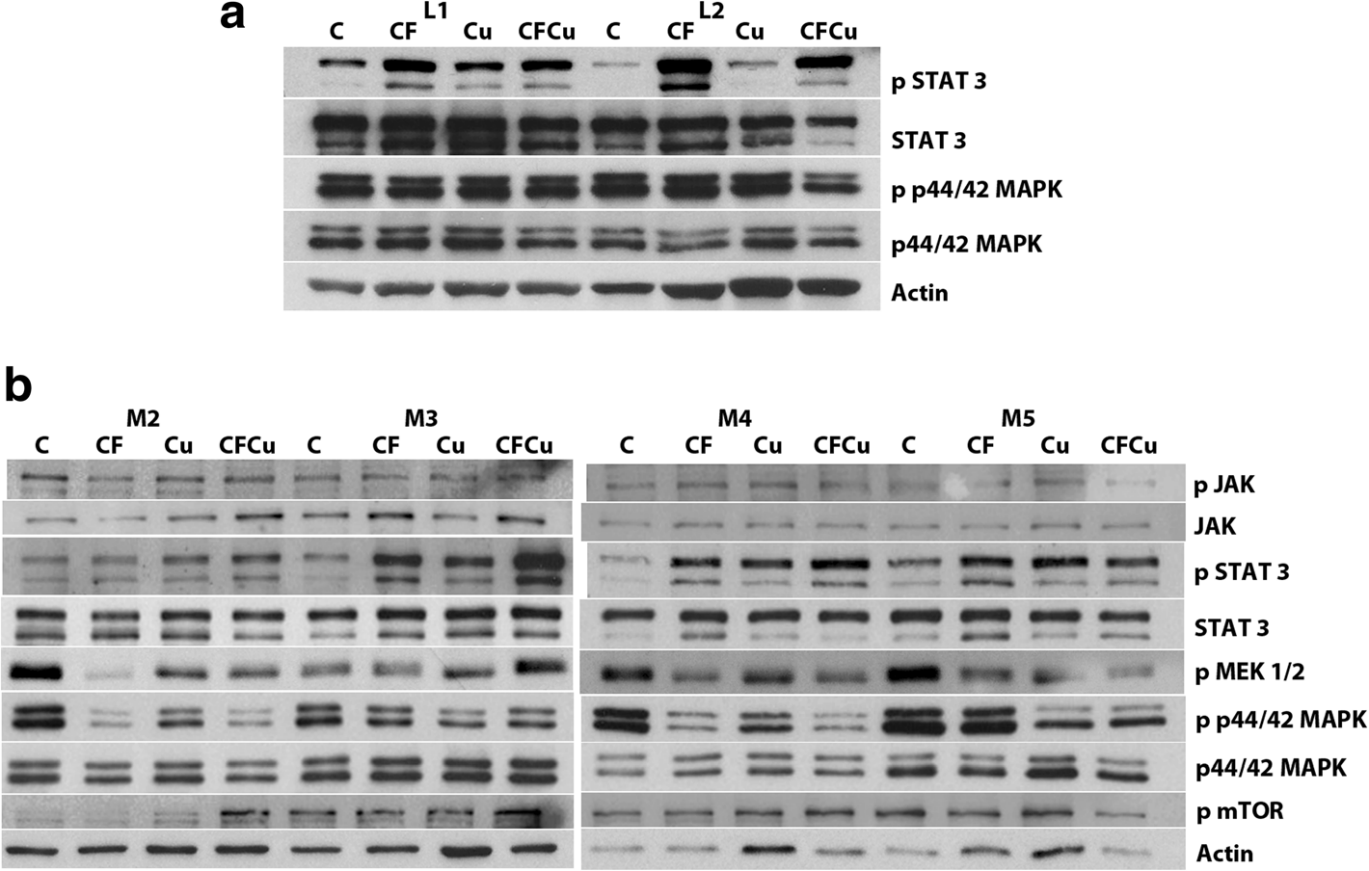
**Western blot analysis of effects of cucurbitacin I on phosphorylation/activation of signaling pathways in human leptomeningeal and meningioma cells. a.** Effects of cucurbitacin I on phosphorylation/activation of STAT3, and p44/42MAPK in human leptomeningeal cells. Leptomeningeal cells from 17 (L1) and 20 wk (L2) were treated with serum free media (C), cerebrospinal fluid (CF) without or with cucurbitacin I (Cu) (250 nM) for 72 hrs. Cerebrospinal fluid stimulated STAT3 phosphorylation. Cucurbitacin I had no effect on basal STAT3 phosphorylation but co-administration with cerebrospinal fluid blocked cerebrospinal fluid stimulation of STAT3 phosphorylation in each. **b.** Effects of cucurbitacin I on phosphorylation/activation of JAK, STAT3, pMEK1/2, p44/42MAPK and mTOR in meningioma cells. WHO grade I (M2 and 3) and (B) WHO grade II (M4 and 5) meningiomas were treated with serum free media (C), cerebrospinal fluid (CF) without or with cucurbitacin I (Cu) (250 nM) for 72 hrs. In the grade I meningiomas, cerebrospinal fluid stimulated phosphorylation of STAT3 and decreased it in MEK1/2. Cucurbitacin I had no effect. In the grade II meningiomas, cerebrospinal fluid stimulated STAT3 phosphorylation in all and reduced phosphorylation of MEK1/2 in all and p44/42MAPK in one. Cucurbitacin reduced basal p44/42 MAPK phosphorylation.

Cerebrospinal fluid had no effect on JAK1 phosphorylation in any of the meningioma cells studies (M2-M6) but stimulated STAT3 phosphorylation in all 3 leptomeningeal cultures and meningioma cells M1, M3–M6. Cucurbitacin I decreased STAT3 phosphorylation in L1-L3. Co-administration of cerebrospinal fluid and cucurbitacin I decreased phosphorylation in M5. In M4 an increase was seen with co-administration. Cerebrospinal fluid reduced MEK1/2 phosphorylation in M2, M4-M6. Cerebrospinal fluid with and without cucurbitacin I was also associated with a reduction of MEK1/2 phosphorylation in M2, M4-M6. Cerebrospinal fluid with or without cucurbitacin I also decreased p44/42MAPK phosphorylation in L2, M4-M6 (Figure 4 and data not shown). Cerebrospinal fluid increased Akt phosphorylation in M6. Cucurbitacin I alone and with cerebrospinal fluid had no effect on Akt phosphorylation in M3, 5 and 6. Cerebrospinal fluid, curbitacin I with or without cerebrospinal fluid had no effect on mTOR phosphorylation in M2-M6. Neither cerebrospinal fluid nor cucurbitacin 1 with or without cerebrospinal fluid had an effect on Rb phosphorylation (Figure 4 and data and not shown).

### Effects of cucurbitacin I on caspase 3 activation in meningiomas

Western blots reveal intact native 117 kDa PARP in all of the treatment groups. Cucurbitacin I had no effect on activation of caspase 3 in the L1 and L3 leptomeninges or grade I M1 meningioma cells. Cerebrospinal fluid increased the approximately 78 KDa cleavage product in L3 and M1 (data not shown).

## Discussion

In the present study, curcubitacin I blocked cerebrospinal fluid stimulation of leptomeningeal, WHO grade I and II cell proliferation in each of the primary cell cultures. These findings suggest that cucurbitacin may be useful in treatment of select meningiomas. Cucurbitacin I appears to be a potent inhibitor in meningioma cells and effective at concentrations similar to that effective on nasopharyngeal carcinoma cells [30] and lower than effective on ALK-positive anaplastic large cell lymphoma cells [32] and pancreatic carcinoma cells [29].

In previous studies cucurbitacin selectively inhibited phosphorylation of STAT3 but not ERK 1 or 2 (P44/42 MAPK), JNK-1 or AKT in NIH 3 T3 cells and adenocarcinoma cell lines [26]. Nonetheless, our findings suggest the mechanisms underlying cucurbitacins antiproliferative effects on meningioma cells are more complex. In the leptomeninges and one meningioma, cucrbitacin I’s effects correlated with reduced phosphorylation of STAT3. In one grade II meningioma, co-administration of cerebrospinal fluid and cucurbitacin I appeared to increase STAT3 phosphorylation (M4) but this was in a case showing particularly robust proliferation in response to cerebrospinal fluid which might be harder to block. The meningioma cells also, in some instances, reduced phosphorylation of p44/42MAPK. While p44/42MAPK regulates numerous cellular functions, the findings raise the possibility that it might also participate in cucurbitacin I’s antiproliferative effects in some cells and doses.

Previously we have found that STAT3 phosphorylation/activation was associated with cerebrospinal fluid stimulation of irradiated leptomeningeal cell [39] and meningioma cell proliferation [22,23]. It is also important in meningioma progression to a higher grade [21,40,41]. Cucurbitacin I’s effect on STAT3 appears to be at the level of STAT3 since it had no effect on upstream components of pathways that activate STAT3 particularly JAK1. Cucurbitacin I appears to reduce the level of tyrosine phosphorylation on STAT3 reducing phospho-STAT3 binding to transcription factors [26]. Nonetheless, cucurbitacin I also reduced levels of p44/42MAPK phosphorylation in the leptomeninges, WHO grade I and II meningiomas. Previously we and others have found p44/42MAPK activation important to meningioma cell proliferation [19,42]. Cucurbitacin also blocked PDGF-BB stimulation of leptomeningeal cell proliferation. PDGF-BB activates STAT3 in some cells and also p44/42 MAPK in leptomeningeal cells consequently cucurbitacin I’s effects could reflect inhibition of STAT3 alone or with p44/42 MAPK.

STAT3 is one of a family of proteins with a phosphotyrosine and DNA binding domain that acts as a transcription factor [43-47]. Latent STAT3 is activated by a several growth factor/cytokine receptors that phosphorylate JAK1 in receptor complexes or STAT3 independently resulting in latent cytoplasmic STAT dimerization and translocation into the nucleus where it increases transcription factors promoting cell proliferation [43-46]. Of interest, recombinant IL-6 treatment reportedly had no effect or was inhibitory to WHO grade I meningioma cell proliferation in vitro [21]. Moreover, cerebrospinal fluid activation/phosphorylation of STAT3, at least in human leptomeningeal and meningioma cells, appears to be independent of an IL-6 receptor-JAK-STAT3 pathway [23]. Thus STAT3 activation may be via several cytokine/growth factor receptor/kinases [41] and/or by the MEK-1-MAPK and PI3k-Akt-mTOR pathways [44-47] that we and others have found to be mitogenic to meningioma cells [19,20,42].

Cucurbitacin I effects on meningioma cell apoptosis are uncertain. Curcubitacin I had no detectable effect on cleavage of PARP, a marker of caspase 3 activity. Nonetheless, due to the limitations inherent to using primary cultures and limited amounts of cerebrospinal fluid from patients without neurological disease, our analysis was limited in scope and time points analyzed. In another study, in combination with gemcitabine, cucurbitacin increased PARP cleavage in pancreactic carcinoma cells [29].

Cerebrospinal fluid from multiple different patients stimulated leptomeningeal and meningioma cell proliferation. These findings extend our previous observations that cerebrospinal fluid from a numerous different adults of various ages and both genders has the potential to stimulate leptomeningeal or meningioma cell proliferation under some circumstances in vivo and may participate in the pathogenesis of meningiomas [23]. For example, recently we have found that prior irradiation, a known initiator of meningioma formation, may sensitize leptomeningeal cells to the mitogenic effects of cerebrospinal fluid in some scenarios [39]. These findings underscore the need to clarify the effects of cerebrospinal fluid on meningioma growth and identify chemotherapies that may block any mitogenic effects *in vivo*.

## Conclusions

Cucurbitacin I is a potent inhibitor of meningeal and meningioma cell proliferation that warrants further study as a nonsurgical alternative therapy for meningiomas. While its mechanism of action may be primarily thru inhibition of STAT3 phosphorylation/activation, other mechanisms may contribute to its growth inhibitory effects.

## Competing interests

The authors declare that they have no conflicts of interest in performance or reporting of this work.

## Authors’ contributions

MJ contributed to the design analyzed the data and wrote the paper. MO contributed to the design, performed the assays and edited the manuscript. KW contributed the tissue, design and editing the manuscript. All authors read and approved the final manuscript.

## Pre-publication history

The pre-publication history for this paper can be accessed here:

http://www.biomedcentral.com/1472-6882/13/303/prepub
